# Heterogeneous resource allocation can change social hierarchy in public goods games

**DOI:** 10.1098/rsos.170092

**Published:** 2017-03-08

**Authors:** Sandro Meloni, Cheng-Yi Xia, Yamir Moreno

**Affiliations:** 1 Institute for Biocomputation and Physics of Complex Systems (BIFI), University of Zaragoza, Zaragoza 50009, Spain; 2 Department of Theoretical Physics, University of Zaragoza, Zaragoza 50009, Spain; 3 Key Laboratory of Computer Vision and System (Ministry of Education) and Tianjin Key Laboratory of Intelligence Computing and Novel Software Technology, Tianjin University of Technology, Tianjin 300384, People’s Republic of China; 4 Complex Networks and Systems Lagrange Lab, Institute for Scientific Interchange, Turin, Italy

**Keywords:** public goods games, complex networks, Pareto Law, adaptive dynamics

## Abstract

Public goods games (PGGs) represent one of the most useful tools to study group interactions. However, even if they could provide an explanation for the emergence and stability of cooperation in modern societies, they are not able to reproduce some key features observed in social and economical interactions. The typical shape of wealth distribution—known as Pareto Law—and the microscopic organization of wealth production are two of them. Here, we introduce a modification to the classical formulation of PGGs that allows for the emergence of both of these features from first principles. Unlike traditional PGGs, where players contribute equally to all the games in which they participate, we allow individuals to redistribute their contribution according to what they earned in previous rounds. Results from numerical simulations show that not only a Pareto distribution for the pay-offs naturally emerges but also that if players do not invest enough in one round they can act as defectors even if they are formally cooperators. Our results not only give an explanation for wealth heterogeneity observed in real data but also point to a conceptual change on cooperation in collective dilemmas.

## 1. Introduction

One of the key elements of human and animal societies is the interaction between groups of individuals to achieve a common goal. The study of cooperation and coordination between individuals has always attracted the attention of scientists from very different fields, ranging from biology [1] and sociology [2,3] to economics [4,5]. On the theoretical side, scientists have tackled this problem using the tools offered by evolutionary game theory [6–9], using among others, public goods games (PGGs) [10–13]. PGGs are usually employed to model the behaviour of groups of individuals achieving a common goal. A typical example are storekeepers with shops in the same street. They can collaborate, i.e. invest some money, in improving the street—more parking slots, better lighting, etc.—to get benefits that will be shared by all the stores: i.e. more customers circulating in the street. However, some of them can decide not to contribute to the improvements, saving some money while sharing all the same part of the added value created by the community.

Mathematically, PGGs are usually represented as the *N*-person version of the Prisoner’s Dilemma [14–19], where individuals can decide to contribute (cooperate) or not (defect) to the creation of the public goods and all the participants share the benefits. To model the added value generated by the public goods a multiplicative *synergy* factor *r* is employed. The investments collected from the community are multiplied by *r* to represent the increase in the value of the public good. The resulting benefits are then divided equally between all the participants in the form of a pay-off independently to the contribution invested by each of them. To mimic the effect of evolution and adaptation on the two strategies—cooperate to the creation of the public good or defect taking advantage of others’ contributions—an evolutionary rule is applied to all players simultaneously [6,9,20,21]. Despite its simplicity, this representation shows a very rich behaviour and demonstrated itself able to reproduce important traits of real-world societies.

Recently, the search for more realistic models led to the formulation of several modifications of the traditional set-up of the PGG. In this direction, one of the first steps has been the introduction of a structure in the population to take into account the complex interaction patterns present in real societies [22–24]. Simultaneously, other efforts have been put in mimicking realistic traits of our societies like reputation [25], reward [26,27] and punishment mechanisms [28–32], human mobility [33–40] and different types of social heterogeneity [41–52]. Heterogeneity seems to play a fundamental role in cooperative behaviour—although some results question the role of network heterogeneity [53,54]—and several works have dealt with the effects of allowing an uneven distribution of players’ resources [48–52]. However, even if the latter studies have helped to understand the emergence of cooperation in large groups, key aspects of the organization of human societies and markets still remain unexplained; a relevant example being the typical wealth distribution observed in economic systems, which cannot be obtained within the formalism of classical PGG.

In implementing the classical formulation of PGG on networks Santos *et al.* [23] considered each neighbourhood—a node and its directly connected neighbours—as a group playing one instance of the PGG. Thus, in a network of *N* nodes, each round *N* different games are played simultaneously; one for each possible neighbourhood. Following this recipe, individuals participate in different groups according to the number of their neighbours. A player with *m* neighbours will contribute to *m*+1 distinct games: the one centred in her neighbourhood and the *m* centred on her neighbours’ ones. At the end of each round, when each player collected the pay-off from all the games she played, an evolutionary step take place and players evolve using an evolutionary rule [6,9,20,21]. In this classical formulation each player that decides to cooperate will divide her capital *c* equally between all the groups she participates in. This choice, even being the simplest one, does not reflect typical human behaviour in economic or social systems. In a more realistic scenario investors playing in a market will react to the behaviour of the system investing in markets that offer higher profits and reducing their involvement in losing ones.

**Figure 1:**
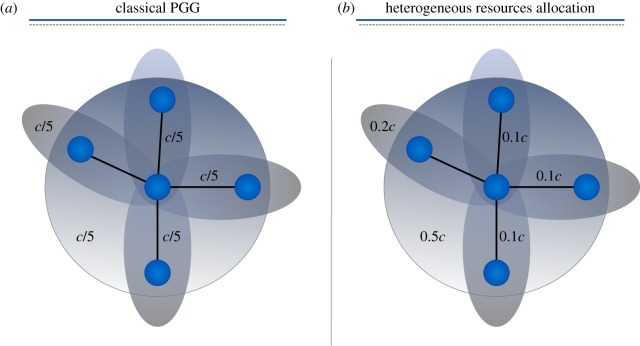
Schematic of PGGs on networks. In the classical framework (*a*) an individual with four neighbours participates in five different games: the one centred on herself and other four centred on her neighbours and divides her contribution *c* evenly. In our model (*b*), contributions can be assigned unevenly according to the pay-off obtained in the previous round, allocating more resources in more profitable games with a higher probability.

Here, we address the previous shortcomings and consider a modification of the classical *N*-person Prisoner’s Dilemma on networks. In our model, players are allowed to distribute their investments unevenly, allocating more resources to profitable games and less in unfavourable ones. We take as starting point the formulation of PGG on networks of Santos *et al.* [23] but, instead of dividing evenly the contribution between the games, we let players decide how to distribute their capital according to what they earned in previous rounds (figure 1). To mathematically model this situation we employ a simple distribution function with a single parameter *α* (see equations (4.1) and (4.2) for details). In this context, *α* can be seen as a modulator of the intensity of this rational behaviour. For *α*=0, we recover the classical formulation where player’s resources are shared evenly between all the games irrespectively of the gains produced by each one. Increasing *α* produces a more aggressive behaviour investing more money in the games that produced the highest revenues in the previous round (see Material and methods for model’s details).

## 2. Results

**Figure 2:**
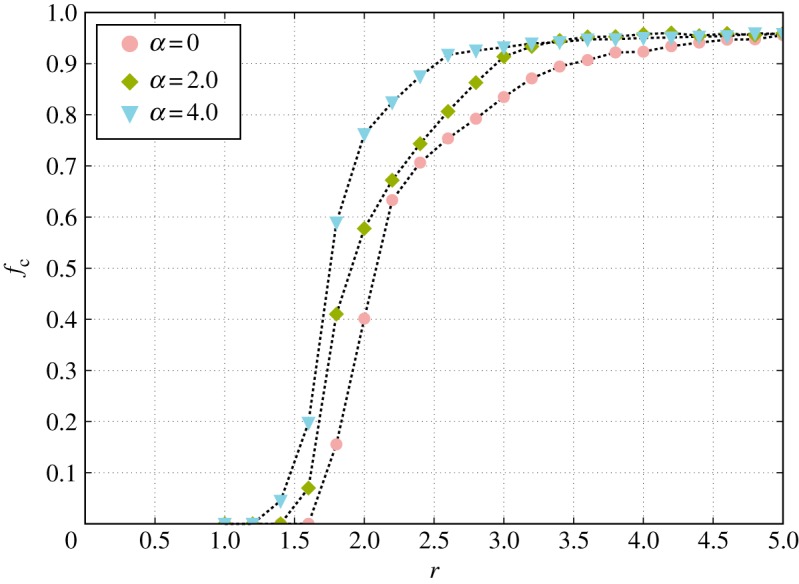
Fraction of cooperators *f*_*c*_ as a function of the enhancement factor *r* for different values of *α*. Results show that there is an increase in the levels of cooperation for highly heterogenous resource allocation (positive values of *α*, see legend). The interaction topology is an uncorrelated scale-free network [55] with exponent *γ*=2.5 and *N*=10^4^ nodes. Each point represents an average over at least 500 runs with randomly chosen initial conditions.

To test our model, we ran extensive computer simulations of our model for different values of the allocation parameter *α*. As substrate for the games, we employed scale-free networks obtained using the uncorrelated configuration model [22,55] with *N*=10^4^ players and minimum degree $k_{\min}=2$ assuring a minimum size for the PGGs of 3 and average degree 〈*k*〉≃4 (see §4.2 for details about networks creation). With this set-up our first analysis shows that, when individuals are allowed to distribute their investments unevenly, an increase in the cooperation level is observed with a shift of the critical synergy parameter *r*_c_ to lower values (figure 2) with respect to the static allocation scheme (*α*=0) and that this increase is more marked for larger values of *α* (i.e. *α*=4.0). Even though these results are consistent with previous studies on similar models [48–52], the mechanisms behind this increase and their consequences on the organization of the system are still unclear.

### 2.1. Microscopic organization

To address these questions, we study the microscopic organization of cooperation in the system. To do so, we focus on the region where cooperation is the dominating strategy for all the values of *α*—in our set-up, given the results of figure 2, *r*>3.5—looking at how individuals assign their capital to the different games.

**Figure 3:**
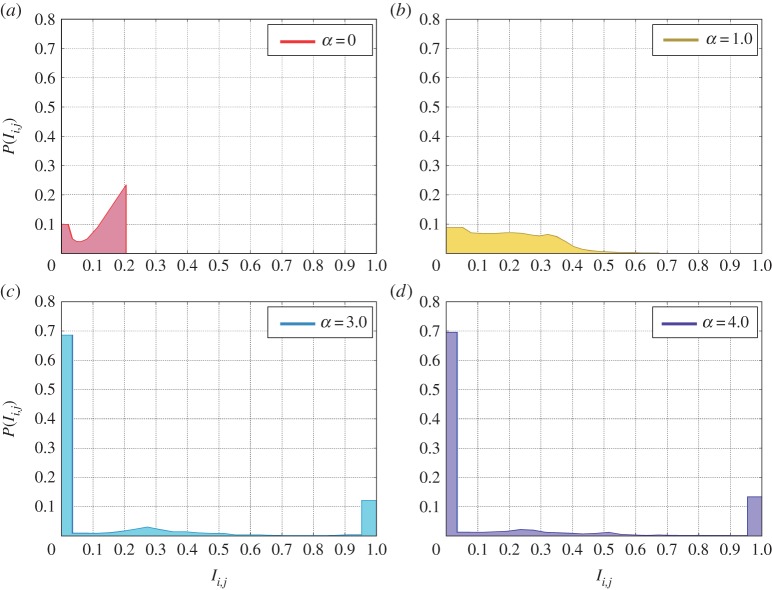
Distribution of the investments *I*_*i*,*j*_ over network’s links for different values of the resource allocation parameter *α* at the steady state. A radical change in the distribution is observed from the static case *α*=0 (*a*), in which the investments follow the degree distribution of the underlying network, to the profit-driven case *α*>0 (*b*–*d*) in which the invested quantity in a game is related to the previously earned pay-off in that game. For dynamic resource allocation and *α*>1.0 (*c*,*d*) a two-peaked distribution appears where players decide to put almost the totality of their resources in one game and invest a minimal quantity in other games. The substrate topology is an uncorrelated scale-free network [55] with exponent *γ*=2.5 and *N*=10^4^ nodes. The synergy factor *r* is set to *r*=4.0 and *c*=1.

Figure 3 depicts how players distribute their investments over the games for all the players once the system reached a stationary state. Here *I*_*i*,*j*_ represents the fraction of player *i*’s capital invested in the game centred on player *j* while *P*(*I*_*i*,*j*_) stands for its distribution over all the players in the system.

For the static resource allocation (*α*=0), the investment distribution clearly follows the degree distribution of the underlying social graph as players can only distribute their contribution evenly between all the games in which they participate. The picture totally changes when we consider a dynamic allocation of the investments. As *α* increases from 0, the investment distribution rapidly become more heterogenous. Beyond *α*=2, two large peaks centred, respectively, at very large (greater than 0.95) and very small (less than 0.05) values of *I*_*i*,*j*_ appear.

**Figure 4:**
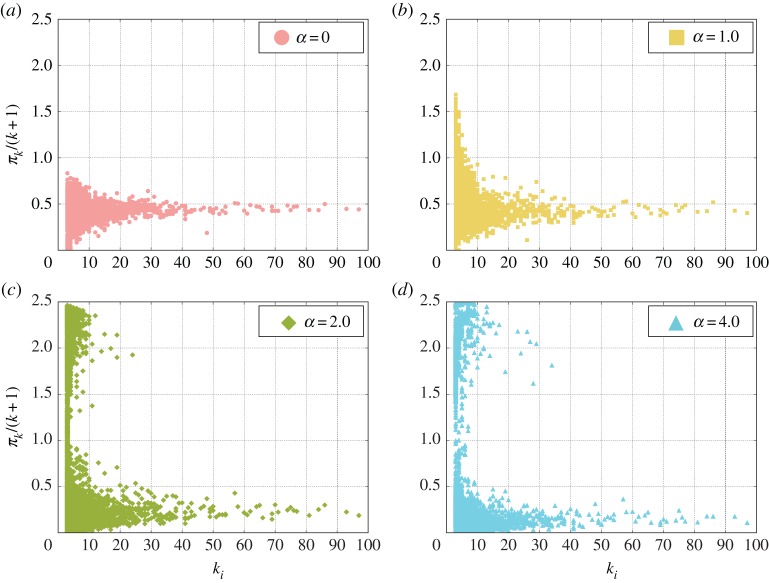
Normalized pay-off *Π*_*k*_/(*k*+1) obtained in the game centred at node *i* as a function of the degree *k* for different values of the parameter *α*. For the static case, *α*=0 (*a*), a low dispersion around a mean pay-off value is observed for all degrees *k*. Increasing *α* leads first to an increase in the normalized pay-off and its dispersion, most notably for small degrees. A further increase of *α* produces a second cloud of points localized at the maximal contribution (e.g. *c*(*r*−1), in this case the maximum value is 2.5 as we have *c*=1 and *r*=3.5) and low degrees (*b*–*d*). Networks parameters are the same as in figure 3.

Results shown in figure 3 suggest that, once players are free to allocate their resources, a very peculiar organization emerges. The peak for large values of the investments indicates that most of the players (almost the totality for *α*≥3.0) allocate the majority of their resources in only one market—the most profitable one—while they distribute evenly between all the other games the remaining part of their capital creating the peak for small values of *I*_*i*,*j*_. The previous findings could explain the observed increase in players’ cooperation [48–52], but it is not the only consequence of the observed investment distribution. Indeed, an established result in PGGs is that the most connected nodes—the hubs—are responsible for the emergence of cooperation and for the production of the majority of the pay-off. However, the results in figure 4 depict a different scenario. If we consider the total normalized pay-off produced in games centred on nodes of degree *k*, *Π*_*k*_/(*k*+1), we find that, in the classical case, it is distributed almost homogeneously among all the degrees, with a mean value around 0.5 (figure 4*a*). However, for *α*>0 strong differences arise. As *α* increases, the distribution of the pay-off for games taking place on low degree nodes starts to become more heterogenous until, finally, for *α*=4.0 (figure 4*d*) a large number of games produce very high pay-offs (note that the maximum pay-off does not depend on *k*, as max{*Π*_*k*_/(*k*+1)}=*c*(*r*−1) and in this case *c*=1 and *r*=3.5 leading to a maximum of 2.5). This means that all the players invested all their contributions in that game. Moreover, with the increase of *α* the average pay-off produced in the hubs decreases substantially.

These results, also in light of the investments’ distribution (figure 3), indicate a profound change in the social structure of the system. While in classical PGG, hubs represent the driving force and the centres where the majority of the wealth is produced, in our model, games on poorly connected nodes are responsible for the creation of the largest part of the public goods. Specifically, our results demonstrate that individuals self-organize in a large number of small-sized clusters formed exclusively by cooperators where all the players invest almost their entire capital. Although our model is a simplified abstraction of economic societies, this peculiar result is also in line with real societies where only a small elite can access highly profitable markets and big investors earn more money participating in several markets at the same time. However, this different organization of cooperators also has other interesting consequences that will be discussed in the next sections.

### 2.2. Wealth distribution

One of the criticisms to the classical *N*-persons’ Prisoner’s Dilemma (also on heterogeneous social structures) is that it fails to reproduce the wealth distribution observed in real economic systems—the so-called Pareto principle [56]—where 80% of the total wealth is generated by 20% of the population. This is mainly due to the fact that, even if games centred on hubs provide higher pay-offs because many players participate in them, they are only a small fraction of the population (surely much less than the 20%) and are not able to significantly change the overall wealth distribution. As in our model cooperators tend to form small but very productive clusters it is interesting to look at the wealth distribution produced for different values of *α*.

**Figure 5:**
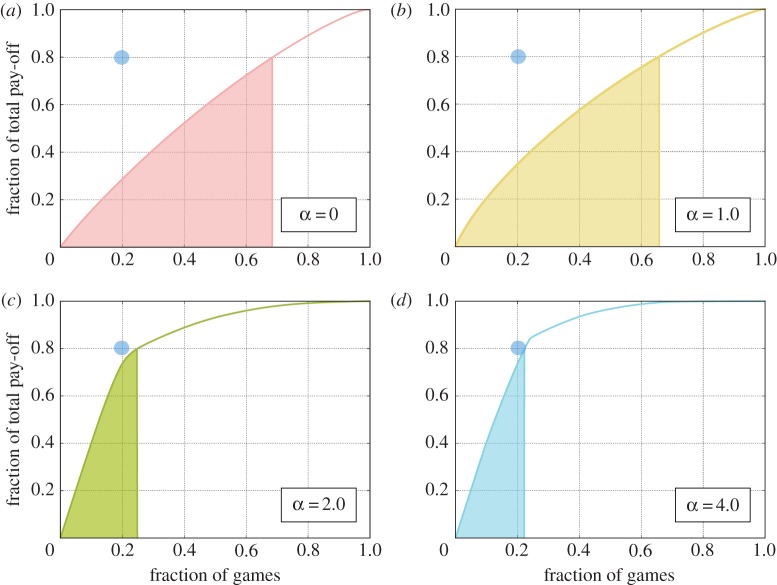
Cumulative fraction of the total normalized pay-off produced in the network as a function of the nodes ranked from the most productive to the less productive ones for several values of *α*. The coloured regions depict 80% of the total wealth and the blue circle represents the optimal value where 80% of the wealth is produced by 20% of the nodes. For the static (*α*=0.0) resource allocation (*a*), more than 60% of the games is needed to produce 80% of the total wealth while for dynamic resource allocation (*α*>2.0) almost 20% of the nodes alone produces 80% of the wealth, resembling a *Pareto Law* [56]. Results represent the average over at least 500 different initial conditions. The other parameters are the same as in figure 3.

The coloured area in figure 5 indicates 80% of the cumulative fraction of the total normalized pay-off produced by nodes ranked from the most to the least productive ones. In the classical PGG, almost 70% of the nodes are required to reach 80% of the wealth while for *α*>1.0 this value reaches approximatively 24% and for higher values of *α* becomes more stable and asymptotically approaches 20% (see the electronic supplementary material). Given that we have not imposed any rule on the PGG other than a stochastic investment mechanism and a replicator-like evolution of the strategies, it can be said that the resulting Pareto Law is obtained from first principles.

### 2.3. Negative links

The uneven investment distribution observed in figure 3 also has another important implication for the games’ dynamics. In fact, we noted that if the contribution of a player *i* in a game is significantly smaller than the average of the other ones, the pay-off obtained by the other players is smaller than what they would obtain if player *i* did not participate, i.e. if the link between *i* and the focal player of the game does not exist. In this context, it is important to note that, even if player *i* is formally a cooperator, for that specific game she is acting as a sort of defector as her presence has the effect of reducing the income of other players. Mathematically, this condition can be represented by the following inequality:

2.1 $$\frac{r}{k_{j}+1}\left(\sum_{l\in \nu_{j}}I_{l,j}(t)s_{l}(t)\right)<\frac{r}{k_{j}}\left(\sum_{l\in \nu_{j}\setminus i}I_{l,j}(t)s_{l}(t)\right),$$

where *ν*_*j*_ represents the neighbourhood of agent *j* (all the first neighbours of agent *j* plus agent *j* itself) while *ν*_*j*_∖*i* stands for the same set excluding player *i*. An important implication of equation (2.1) is that it allows us to classify every directed link according to whether it is verified or not. In this way, we can define a link as *positive* if equation (2.1) is not satisfied—the contribution on the link is large enough to create an added value in the game—or, in the opposite way, a link as *negative* if the contribution of player *i* is small enough to satisfy equation (2.1) implying that the absence of the link would be beneficial for the other players of the game.

**Figure 6:**
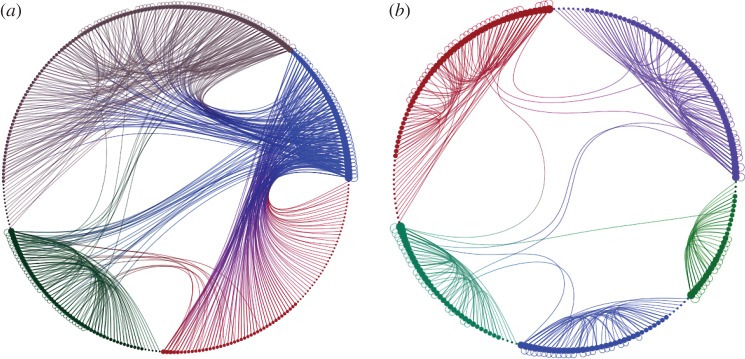
Visualization of the networks obtained considering only *negative* (*a*) and *positive* (*b*) links from the same original network of size *N*=300. The original graph is a scale-free network generated according to the uncorrelated configuration model [55] with *γ*=2.5 and *N*=300 nodes.

By analysing how the two types of links are organized, we can dissect the entire network in two subgraphs: one formed only by negative links and the other containing the positive ones. The analysis of the two networks brings about interesting results. We found that in almost all the realizations the two networks were connected graphs (only in a few cases the positive network presented some isolated nodes) and, more importantly, in all the cases, the positive network had a backbone-like structure with similar topological features of the minimum spanning tree of the original network. On the other hand, the negative network always includes the majority of the links and its structure strictly resembles the original one (the details of the topological analysis and the comparison between the positive network and the minimum spanning tree are given in the electronic supplementary material). Also visually (figure 6) the differences between the two networks is notable, with the positive network formed by long chains of poorly connected nodes resembling the spanning tree. It is worth stressing that this backbone organization of the links at the entire network level spontaneously emerges as a consequence of the self-organization of the players at the local level without any control mechanism.

Finally, it is also noteworthy that even if all the presented results have been obtained in the so-called *fixed cost per player* (FCP) paradigm where each individual has the same capital *c*, our results qualitatively hold also for the opposite case of a *fixed cost per interaction* paradigm, where players have a capital *c* for each game (link) in which they participate (see the electronic supplementary material). In addition, to further prove the robustness of our findings we also test different evolutionary rules beyond the finite size equivalent of the replicator dynamics like unconditional imitation [57–59] and the Fermi rule [57–60]. In all the cases, the results reveal the same qualitative behaviour and very small quantitative differences (see electronic supplementary material for details).

## 3. Conclusion

Even though heterogeneity has been recognized as one of the most effective mechanisms to favour cooperation in evolutionary games [41–52] some of its consequences still remain uncovered.

Aimed at shedding light on the organization of cooperation in PGGs, in this paper, we have focused on a different rule for investments that allows players to allocate their resources unevenly. Although this modification might appear not significant, it leads to a substantial change that can help us explain social and economic hierarchies observed in our complex society. Specifically, despite of its simplicity, our model offers a first-principled explanation of the Pareto Law for wealth distribution [56]. This result is a direct consequence of the bimodal distribution of investments observed in figure 3, resulting from a behaviour in which players invest the majority of their contribution in one game creating small productive clusters of nodes. Although the emergence of those clusters is responsible for the heterogeneity observed in the cumulative pay-off distribution (figure 5), the reason why this 80–20 rule is so stable for a large range of values of *α* is still unclear and surely deserve further studies.

The heterogenous pay-off distribution is not the only consequence of the uneven investments allocation. Analysing how players distribute their contribution, we also found that if the investment on one link is below the threshold given by equation (2.1), the presence of the link is detrimental for the other players leading to a lower pay-off. This result imposes a change in how we think about cooperation in evolutionary game theory as one player can act as a cooperator in one game (link) and as a defector in others. This concurrent behaviour makes it more meaningful to speak in terms of cooperation in games rather than of cooperators. That is, it suggests that in some contexts, the term cooperation should be carefully used, and that it may be more natural to change the reference of cooperation from players to games.

It is also important to note that the presence of these so-called *negative* links is not an exclusive feature of our model, but it represents a usual situation also in the classical formulation of PGG on heterogeneous networks with the FCP paradigm. To clarify this point, it is instructive to focus on a toy example. Let us consider a simple network composed by a ring of *n* nodes in which each node is connected only to its two nearest neighbours and to a hub placed at the centre of the ring, i.e. a wheel-like configuration. In this case, the hub will have degree *n* while the other nodes in the ring have degree 3. In the FCP set-up, the hub will be involved in *n*+1 games and its contribution in each game will be *c*/(*n*+1) while the other nodes will participate in four different games and contribute to each *c*/(3+1). It is straightforward to demonstrate that in the games centred on the leaves for *n*>4 equation (2.1) holds for all possible values of *r* and *c*. In this case even if the hub is formally a cooperator its presence reduces the pay-off obtained by all the other nodes in the ring.

Equation (2.1) allowed us also to classify contributions as *negative* or *positive* and to split the original network into two layers each one made up of links of the same type. We found that not only players self-organize their positive links to create highly productive groups but also at a higher level they tend to form a backbone of the entire network. The structure we found strictly resembles the minimum spanning tree of the original interaction network and in most of the cases covers more than 90% of the agents in the system.

Finally, taken together, our findings not only explain the increase in cooperation observed in previous studies [48–52] but also can help to understand the basis behind the heterogeneity in wealth distribution observed in almost all human societies and give a hint about the organization and functioning of large economic systems. Our results also impose a substantial change in our idea of cooperation in evolutionary game theory as they demonstrate that also in the classical formulation of PGGs on networks players can act as cooperators and defectors at the same time.

## 4. Material and methods

We consider a PGG on networks [23] with a dynamical resource allocation scheme that allows individuals to invest higher quantities in profitable groups and reserve their resources from unfavourable ones. Each node *i* of the network is considered as a player participating in *k*_*i*_+1 different PGGs with its neighbours. Participating in a PGG round each individual can decide to contribute (cooperate) a part of its resources or act as free-rider (defect) and not contribute to the public good. Following the formalism for PGGs on networks introduced in [23] two different contribution schemes are possible, the so-called FCP and *fixed cost per game* (FCG).

For the FCP scheme the total amount of resources for each round is fixed to *c* and equal for all the players, i.e. all the players have access to the same capital irrespective of the number of games in which they are participating. In the FCG scheme, each player can dispose of a capital *c* for each game she is involved in, that is, the total capital for each node depends upon the number of games she plays. A node of degree *k* participates in *k*+1 games and has access to a capital of (*k*_*i*_+1)*c*. In the classical formulation for both the FCP and FCG schemes, cooperators divide their contributions equally between all the games they are involved in; resulting in an equal contribution of *c*/(*k*+1) for the FCP and *c* for the FCG.

In our model, in the case of cooperation, the contribution of each agent in a game is calculated dynamically and depends on the pay-off obtained by the player in the previous round of the game. The investment of player *i* at time *t*+1 in the game where node *j* is the focal player is defined as *I*_*i*,*j*_(*t*+1) and for the FCP reads as:

4.1 $$I_{i,j}(t+1)=\frac{ce^{\alpha \Pi_{i,j}(t)}}{\sum_{l\in \nu_{i}}e^{\alpha \Pi_{i,l}(t)}},$$

while in the FCG the same quantity is multiplied by the number of games *k*+1:

4.2 $$I_{i,j}(t+1)=(k_{i}+1)\frac{ce^{\alpha \Pi_{i,j}(t)}}{\sum_{l\in \nu_{i}}e^{\alpha \Pi_{i,l}(t)}},$$

where in all the cases *Π*_*i*,*j*_(*t*) is the pay-off obtained by agent *i* in the game centred on node *j* at the previous time step, *ν*_*i*_ is the set of all the first neighbours of node *i* plus node *i* itself and *α* is a parameter that allows us to differentiate between a static and homogeneous resource allocation (*α*=0) and heterogeneous distributions where higher resources are invested in best performing games (*α*>0). At time *t*=0, as all the previous pay-offs are set to zero, the contribution is divided evenly between all the games.

In this setting, the pay-off *Π*_*i*,*j*_(*t*) of player *i* in the game where *j* is the focal player can be calculated as:

4.3 $$\Pi_{i,j}(t)=\frac{r}{k_{j}+1}\left(\sum_{l\in \nu_{j}}I_{l,j}(t)s_{l}(t)\right)-I_{i,j}(t)s_{i}(t),$$

where *r* is the synergy factor and *s*_*x*_(*t*) is a dichotomous variable representing cooperation *s*_*x*_(*t*)=1 and defection *s*_*x*_(*t*)=0, respectively. Summing over all the games in which player *i* participates the total pay-off earned by *i* at time *t* reads as:

4.4 $$\Pi_{i}(t)=\sum_{j\in \nu_{i}}\Pi_{i,j}(t).$$

At the end of each round players update synchronously their strategies according to the finite population equivalent of the replicator dynamics [58,59]. Each player *i* selects with uniform probability one of her neighbours *j* and compares their respective pay-offs. If *Π*_*i*_(*t*)≥*Π*_*j*_(*t*) the player will keep her strategy in the next time step otherwise, with probability P(i→j) player *i* will copy the strategy of *j*. We can calculate P(i→j) as:

4.5 $$P(i\to j)=\frac{\Pi_{j}(t)-\Pi_{i}(t)}{M},$$

where *M* is a normalization factor defined as the maximum possible pay-off difference between two players in the network assuring that 0≤P(i→j)≤1.

### 4.1. Numerical set-up

Numerical results presented in the text are the average of at least 500 independent runs with randomly chosen initial conditions. At the beginning of each run players are assigned randomly one of the two available strategies (cooperate or defect) with probability 0.5. The average density of cooperators and the other quantities considered are evaluated at the stationary state after a sufficiently long relaxation time (usually 5×10^4^ time steps) and then averaged over additional 10^3^ steps.

### 4.2. Networks

As a substrate we employ scale-free networks generated according to the uncorrelated configuration model [22,55]. In the configuration model each one of the *N* nodes is assigned a desired degree drawn from a distribution—in this case, a power law of the form *P*(*k*)∼*k*^−*γ*^—and a number of stubs (half edges) equal to its degree is attached to each node. Stubs are then connected randomly to create the network. This allows to create random networks following a given degree distribution. For long-tailed distributions, to avoid structural correlations [55] that can alter the dynamic behaviour of the system, a limit in the maximum possible degree is imposed: $k_{\max}=N^{1/2}$. Presented results have been obtained using different realizations of scale-free networks of size *N*=10^4^ and exponent *γ*=2.5 resulting in a mean degree 〈*k*〉≃4 (for results with different networks structures, systems sizes and *γ* exponents see the electronic supplementary material). In all the cases, the minimum degree has been kept to $k_{\min}=2$ to guarantee a minimum size for the PGGs of three players assuring that all the games are proper *N*-players PGGs instead of two-players Prisoner’s Dilemmas.

## Supplementary Material

Supporting Information

## Data accessibility

Robustness analysis for all the results presented in the main text is available in the electronic supplementary material.
