# Supplementary material for: Heterogeneous resource allocation can change social hierarchy in public goods games
Source: R Soc Open Sci. 2017 Mar 8;4(3):170092. doi: 10.1098/rsos.170092 (PMC5383863; doi:10.1098/rsos.170092)
Supplement: Supporting Information [file rsos170092supp1.pdf]

Supporting Information: Heterogeneous resource allocation can change social hierarchy in public goods games

Sandro Meloni[1,2], Cheng-Yi Xia[3], and Yamir Moreno[1,2,4]

[1]Institute for Biocomputation and Physics of Complex Systems (BIFI), University of Zaragoza, 50018 Zaragoza, Spain

[2]Department of Theoretical Physics, University of Zaragoza, 50009 Zaragoza, Spain

[3]Tianjin Key Laboratory of Computer Vision and System (Ministry of Education) and Key Laboratory of Intelligence Computing and Novel Software Technology, Tianjin University of Technology, Tianjin 300384, P.R.China

[4]Institute for Scientific Interchange, ISI Foundation, Turin, Italy.

1 Robustness analysis

In this section we demonstrate the robustness of our findings against the three main components of our model: the implementation of public goods games on networks, the evolutionary rule used and the structure of the underlying interaction graph. We also test the influence of the two game parameters: the synergy factor r and the investment allocation parameter α.

1.1 Public goods paradigm

In the modeling of cooperators' contribution in public goods games on networks two different choices are possible. In the so-called *fixed cost per player* (FCP) paradigm each cooperator has the same quantity c independently of the number of games in which she participates and her contribution is divided between all of them, while in the *fixed cost per game* (FCG) the contribution is proportional to the number of games played. In the main text, we presented results for the FCP setup, in which players' investments were given by Eq. (2) in the main text. In this

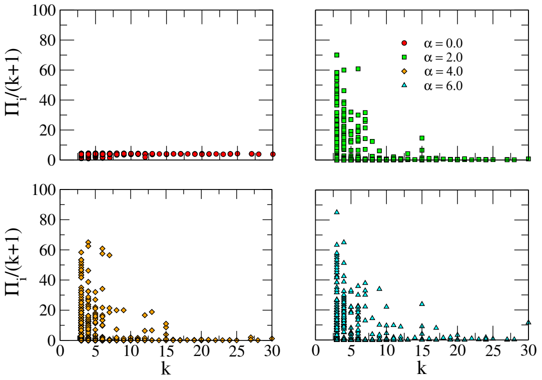

Figure 1: Normalized payoff $\Pi_k/(k+1)$ obtained in the game centered at a node of degree k in the *fixed cost per game* paradigm for different values of $\alpha = 0, 2.0, 4.0, 6.0$ and $r = 6.0$. The interaction graph is an uncorrelated scale-free network with $N = 10^3$ nodes and $\gamma = 2.7$.

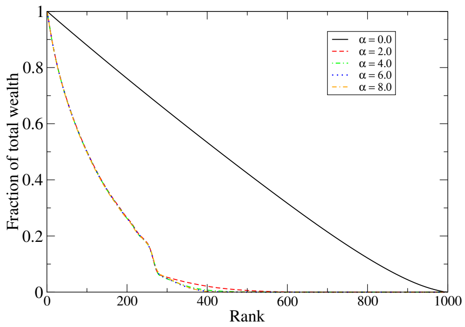

Figure 2: Cumulative fraction of the total normalized payoff produced in the network as a function of the nodes, ranked by the most productive to the less productive ones in the *fixed cost per game* for different values of α and for $r = 6.0$. The interaction graph is an uncorrelated scale-free network with $N = 10^3$ nodes and exponent $\gamma = 2.7$.

section, we demonstrate that our results also hold for the FCG case, in which the investment distribution follows Eq. (3) instead. Fig. 1 shows the normalized payoff ($\Pi_k/(k+1)$) of games centered on nodes of degree k in the FCG paradigm. Although in this case the normalized payoff is not bounded, we found the same qualitative behavior of Fig. 4 of the main text, with a small dispersion around the mean value for the uniform investment allocation ($\alpha = 0$) and a strong heterogeneity for the small nodes for higher values of α.

A similar confirmation can be found if we rank the games from the most to the least remunerative (Fig. 2) and calculate the cumulative fraction of normalized payoff produced in each game. As for the FCP setting, increasing α leads to strong differences between the games and we recover a Pareto-like distribution for the wealth produced in the system although, in this case, the strong differences in individuals' total contributions (that can vary more than two orders of magnitude) generate a sharper drop at the end of the curves. Finally, it's also important to notice that results of Figs. 1 and 2 have been obtained for a wide range of values of α ($0 \leq \alpha \leq 8$) and $r = 6.0$ demonstrating also the stability of our findings with respect to these parameters.

1.2 Effects of evolutionary rule

Another key ingredient in the implementation of PGGs on networks is the rule used to represents the evolutionary and selection process behind the evolution of strategies. In the literature several rules have been proposed with the aim of covering different selection processes for the evolution. In the main text we conducted our analysis focusing on one of the most common rule used in evolutionary game theory, the finite population equivalent of the *replicator dynamics*, where the distribution of each strategy is included in the fitness function. In this section we demonstrate that our main findings also hold for two other common rules employed in the literature: a *Fermi* process and *Unconditional Imitation*.

1.2.1 Fermi process

Along with the *replicator dynamics* another common choice in evolutionary game theory is to relate the adoption probability of a strategy to the payoff it generates. In this context a *Fermi* process is usually employed to link the adoption probability to the difference between the payoff obtained by agents i and j in a non-linear way:

$$P(i \to j) = \frac{1}{1 - e^{-K(\Pi_j(t) - \Pi_i(t))}}, \qquad (1)$$

where $\Pi_i(t)$ and $\Pi_j(t)$ stands for the payoff obtained by agents i and j respectively and K represents the selection intensity.

To test the robustness of our results also in this case we run the entire analysis we made in the main manuscript with the Fermi rule as evolutionary rule in the *fixed cost per player* paradigm. In Fig. 3 we study the investments distribution $P(I_{i,j})$ over the games for $r = 4$, $\alpha = 0, 1.0, 2.0, 4.0$ and a selection intensity of $K = 0.1$ finding the same peaked distribution we find with the finite population equivalent of the *replicator dynamics*. The same applies also for the other results we presented in the main text: the

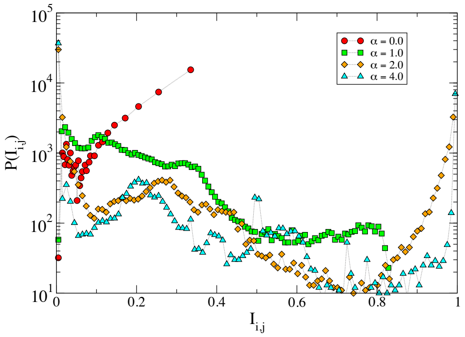

Figure 3: Distribution of the investments $I_{i,j}$ over network's links for different values of the parameter α at the steady state in the *fixed cost per player* using the *Fermi* evolutionary rule with $K = 0.1$ on Scale–Free networks with with $N = 10^4$ nodes and exponent $\gamma = 2.5$.

normalized payoff generated on games of size $k+1$ and the cumulative payoff generated in the network. Fig. 4 and Fig. 5 show no substantial differences with the results presented in the main text with the same parameters and a selection intensity of 0.1.

1.2.2 Unconditional imitation

In the *Unconditional Imitation* (UI) framework agents are supposed to be fully rational and no errors are introduced in the process. In this deterministic scenario each player compares her payoff against her best performing neighbour. If the neighbour has a higher payoff she will adopt the same strategy. As for the other cases we run extensive numerical simulations with the same setting as in the main text with the only exception of the evolutionary rule that in this case is an UI. Fig. 6 shows the distribution $P(I_{i,j})$ of the investments $I_{i,j}$ over the links of a scale-free interaction network at the steady state for $r = 6$. Results confirm our main finding that for $\alpha > 1.0$ the distribution has two marked peaks at very low and very high values and it is almost zero for intermediate values. Also the other two main results are confirmed in this setting as showed in Fig. 7 for the normalized

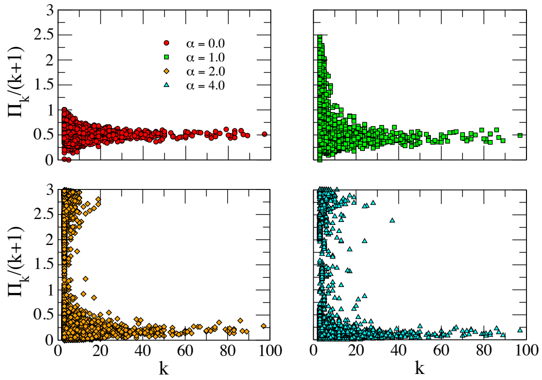

Figure 4: Normalized payoff $\Pi_k/(k+1)$ obtained in the game centered at nodes of degree k in the *fixed cost per player* scenario for different values of $\alpha = 0, 1.0, 2.0, 4.0$ and $r = 4.0$ using the *Fermi* evolutionary rule with $K = 0.1$ on Scale–Free networks with with $N = 10^4$ nodes and exponent $\gamma = 2.5$.

payoff generated by different games and in Fig. 8 for the distribution of the cumulative fraction of the normalized payoff produced in the network with a high heterogeneous distribution of the wealth.

1.3 Effects of network topology

Another fundamental point to demonstrate the robustness of our findings is to check the effect of the interaction network that shapes the size and structure of the different games. In the main text, we presented results obtained using different realizations of uncorrelated scale free networks created via the configuration model with exponent $\gamma = 2.5$ and $N = 10^4$ nodes. In section 1.1, while shifting from FCP to FCG we also changed the size ($N = 10^3$ nodes) and the exponent ($\gamma = 2.7$) of the network leading to the same qualitative results.

In this section, in order to discard that the heterogeneity in the investments and in the payoffs could be a consequence of the heterogeneous degree distribution of the underlying network, we test our model

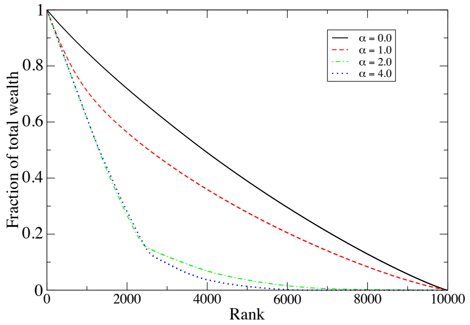

Figure 5: Cumulative fraction of the total normalized payoff produced in the network as a function of the nodes, ranked by the most productive to the less productive ones in the *fixed cost per player* for different values of α and for $r = 4.0$ using the *Fermi* evolutionary rule with $K = 0.1$. The interaction graph is an uncorrelated scale-free network with $N = 10^4$ nodes and exponent $\gamma = 2.5$.

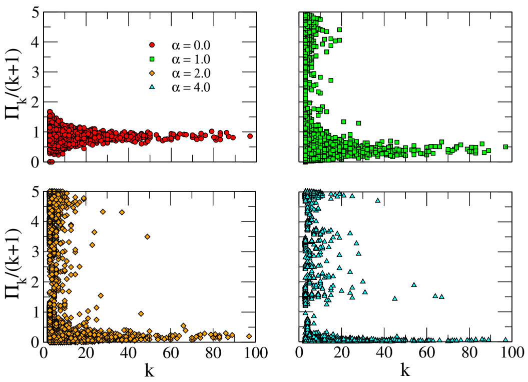

Figure 7: Normalized payoff $\Pi_k/(k+1)$ obtained in the game centered at nodes of degree k in the *fixed cost per player* scenario for different values of $\alpha = 0, 1.0, 2.0, 4.0$ and $r = 4.0$ using *unconditional imitation* as evolutionary rule on a Scale–Free network with with $N = 10^4$ nodes and exponent $\gamma = 2.5$.

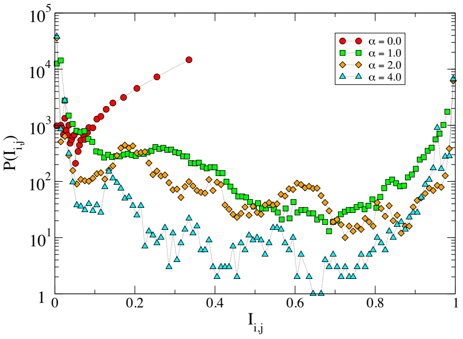

Figure 6: Distribution of the investments $I_{i,j}$ over network's links for different values of the parameter α at the steady state in the *fixed cost per player* using *unconditional imitation* as evolutionary rule on a Scale–Free network with with $N = 10^4$ nodes and exponent $\gamma = 2.5$.

using an Erdös-Rényi random graph with $N = 10^3$ nodes and average degree $\langle k \rangle = 5$ in the fixed cost per player (FCP) scenario. Fig. 9 demonstrates that the distribution of the investments $I_{i,j}$ over the links of the homogenous graph behaves exactly as in the case of the scale-free network with two large peaks for very low and very high values of $I_{i,j}$ and for $\alpha > 0.0$. We also checked for the robustness of the two other results reported in the main paper: the heterogeneity of the normalized payoff and the Pareto-like distribution of the wealth produced collectively. In Fig. 10 we show the normalized payoff of games centered on nodes of degree k for an homogenous network and 4 different values of α. Also in this case the results strictly resemble what we found for the heterogenous networks, i.e., a small dispersion for the static investment allocation and a much wider distribution with few games with the maximum possible payoff for the dynamic allocation ($\alpha > 0.0$). Our results are also confirmed if we look at the cumulative fraction of the capital invested in the network (Fig. 11) characterized by the same Pareto-like distribution we found for the scale-free networks.

Finally, to check the effects of possible dynamical

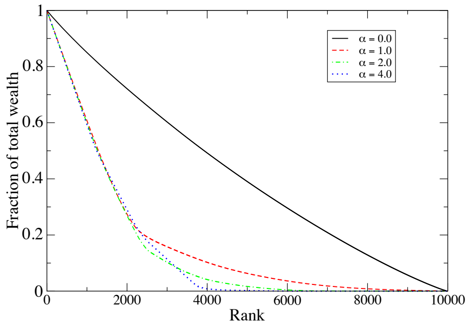

Figure 8: Cumulative fraction of the total normalized payoff produced in the network as a function of the nodes, ranked by the most productive to the less productive ones in the *fixed cost per player* for different values of α and for $r = 4.0$ using *unconditional imitation* as evolutionary rule. The interaction graph is an uncorrelated scale-free network with $N = 10^4$ nodes and exponent $\gamma = 2.5$.

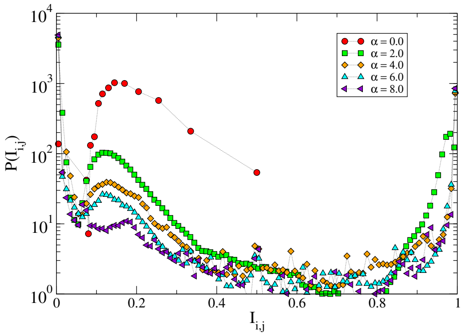

Figure 9: Distribution of the investments $I_{i,j}$ over network's links for different values of the parameter α at the steady state on an Erdös-Rényi graph with $N = 10^3$ nodes and average degree $\langle k \rangle = 5$.

correlations we ran our model on a 2D regular lattice with $N = 2.5 \cdot 10^3$ nodes, $\langle k \rangle = 4$ and periodic boundary conditions, finding a very good agreement with the results for the Erdös-Rényi graphs (not showed).

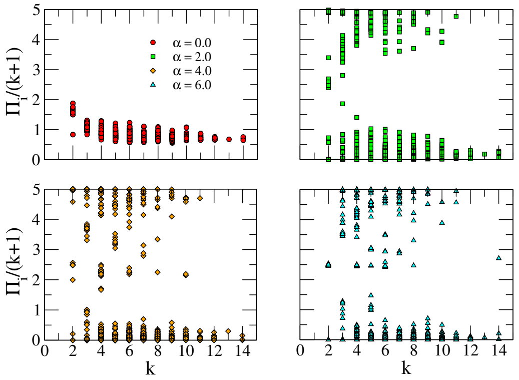

Figure 10: Normalized payoff $\Pi_k/(k+1)$ obtained in the game centered at nodes of degree k in the *fixed cost per player* scenario for different values of $\alpha = 0, 2.0, 4.0, 6.0$ and $r = 6.0$ on a Erdös-Rényi graph with $N = 10^3$ nodes and average degree $\langle k \rangle = 5$.

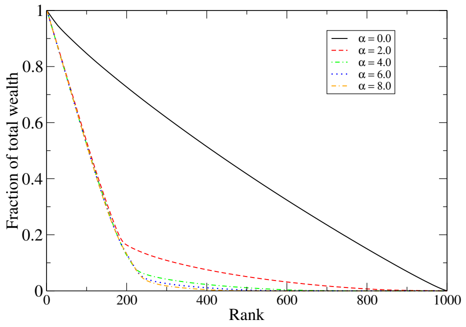

Figure 11: Cumulative fraction of the total normalized payoff produced collectively as a function of the rank of the nodes (from most productive to the less productive ones), in the *fixed cost per player* configuration and for different values of α. $r = 6.0$ and the substrate network is an Erdös-Rényi graph with $N = 10^3$ nodes and average degree $\langle k \rangle = 5$.

	Original	Negative links	Positive links	Spanning Tree
Nodes (1st conn.comp.)	10^4	10^4 (9973.7)	10^4 (7414)	10^4
Links (1st conn.comp.)	20671	20134.54 (19706.2)	10289.25 (9408.03)	9999
# Connected Realizations	-	13	4	100
# Conn. Comps.	1	4.89	676.41	1
$\langle d \rangle$ (1st conn.comp.)	5.09	- (5.15)	- (11.28)	19.84
Diameter (1st conn.comp.)	14	- (15.86)	- (37.93)	57
Clustering Coeff.	0.00389	0.00383	0.0000705	0.0
Transitivity	0.00315	0.00312	0.00000795	0.0
# Triangles	256	254.474	0.67	0
# Cycles	10618	9733.55	1537.65	0
Mean cycle length	282.89	261.244	429.552	0

Table 1: Average topological features of the four considered networks. *# Connected Realizations* stands for the number of realizations ended with a connected network and $\langle d \rangle$ as the average path length. The game parameters are $\alpha = 2.0$ and $r = 4.0$. The original network is a scale-free graph generated according to the uncorrelated configuration model with $N = 10^4$ nodes and exponent $\gamma = 2.5$.

	Original	Negative links	Positive links	Spanning Tree
Nodes (1st conn.comp.)	10^4	10^4 (9602.39)	10^4 (8822.14)	10^4
Links (1st conn.comp.)	18136	15861.6 (14892.78)	11321.2 (9563.13)	9999
# Connected Realizations	-	15	6	100
# Conn. Comps.	1	50.3	422.117	1
$\langle d \rangle$ (1st conn.comp.)	5.73	- (5.88)	- (10.67)	19.84
Diameter (1st conn.comp.)	14	- (16.24)	- (31.67)	57
Clustering Coeff.	0.0019	0.0019	0.00029	0.0
Transitivity	0.0020	0.001949	0.00038	0.0
# Triangles	98	93.28	4.33	0
# Cycles	8137	6260.19	2500.01	0
Mean cycle length	384.99	270.855	527.106	0

Table 2: Average topological features of the four considered networks. The game parameters are $\alpha = 4.0$ and $r = 6.0$. The original network is a scale-free graph generated according to the uncorrelated configuration model with $N = 10^4$ nodes and exponent $\gamma = 2.7$.

2 Positive and Negative networks analysis

In the main text, we have discussed a method that allows distinguishing between links (collaborations) that offer a real advantage to the games and the ones in which the investment is not enough to increase the revenue of the other participants. Using Eq. (2.1) of the main text, we can classify the links in two categories: *Positive* links, in which the contribution is high enough as to increase the payoff of all the group players; and *negative* links, in which the investment is much lower than the average of the other contributions, which leads to a lower collective payoff with respect to the case in which the link would not exist. Splitting the original network in two subnetworks according to the type of the links, we also showed that the so-called *positive* network can be seen as a backbone of the network that has strong similarities with its minimum spanning tree. On the contrary, the network made up of *negative* links resembles the original one. The rationale of these patterns is that, as players invest the majority of their capital in just one game, the entire system self-organizes to create a giant cluster connected only by such links.

To support the previous hypothesis, we next analyze the structure of the two networks and compare them with the minimum spanning tree calculated over the original one. However, due to the stochastic nature of both the model – i.e., in the initial conditions and in the evolutionary rule – and the calculation of the minimum spanning tree, a strict comparison at the link level is impossible and we must rely on the average features of different realizations of the networks and the minimum spanning tree. To this end, we run our model 10^2 times on a single scale-free network with random initial conditions and, for each run, we calculate the positive and negative links. For the same network we also extract the minimum spanning tree with 10^2 different initial seed nodes.

In Tables 1 and 2 we report the average topological features obtained over all the realizations of the four networks for different values of α and r. While both the positive and negative networks in most of the cases are not connected, they both show a very large giant component. Most notably, the number of links of the two networks is rather different: while the negative backbone contains the majority of the links and almost match those of the original graph; the positive network has, on average, exactly one link per node. At this point, it is important to notice that even if the original network is undirected, the interactions are directed so the number of links of the positive and negative network can differ from the original one. The very low number of links of the positive network leads to a higher average path length and diameter of the network, whose values are half way between the original network and the corresponding average of the minimum spanning tree. On the contrary, the negative network only shows a small variation with respect to the original one.

Finally, the last condition needed to fully characterize a minimum spanning tree of a network, along with the number of nodes and links, is the absence of circles of any length. Studying the average clustering coefficient and the transitivity we obtain a quantitative measure of the number of triangles in the network; while the number of circles of higher order can be counted as well. Also in this case the positive network resembles the spanning tree with no triangles and an order of magnitude less circles of higher order than the negative and the original networks. To conclude our analysis, we present an example of the degree distribution of the four graphs (Fig. 12) finding that, although for very low degrees some differences are present (probably due to the fact that the positive network is disconnected), in general the positive network and the spanning tree show a similar structure. These latter results are corroborated if we run a 2-Samples Kolmogorov-Smirnov test of the degree distributions of the four networks. The comparison between the degree distribution of the positive network against the negative one leads to reject the hypothesis that the networks share the same degree distribution ($p < 0.001$) while the comparison between the negative network and the original one produces a p-value $p \simeq 0.12$. The comparison between the positive network and a spanning tree does not bring convincing results as the K-S test leads to $p < 0.05$ but this could be due to the differences for low degree nodes highlighted in Fig. 12.

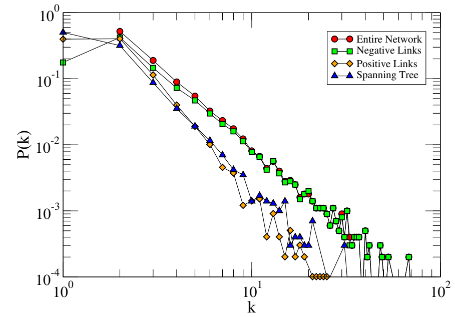

Figure 12: Degree distributions of the four networks: the original network *(circles)*, the one including only the negative links *(squares)*, the one including only the positive links *(diamonds)*, and one realization of the spanning tree of the original network *(triangles)*. The original network has $N = 10^4$ nodes and has been generated via the uncorrelated configuration model.
